# Recto-Vaginal Fistulæ, and Fistulæ about the Anus in Woman

**Published:** 1895-01

**Authors:** A. Lapthorn Smith

**Affiliations:** B. A., M. D., M. R. C. S., Eng., F. O. S., London; Fellow of the American Gynæcological Society; Gynæcologist to the Montreal Dispensary; Surgeon to the Woman’s Hospital, Montreal, Canada; 250 Bishop Street


### RECTO-VAGINAL FISTULÆ AND FISTULÆ ABOUT THE ANUS IN WOMEN.

BY A. LAPTHORN SMITH, B. A., M. D., M. R. C. S., ENG., F. O. S., LOND

Fellow of the American Gynæcological Society; Gynæcologist to the Montreal Dispensary; Surgeon to the Woman's Hospital, Montreal, Canada.

The evolution of obstetrics is gradually bringing about a distinct change in the nature of the injuries to the pelvic contents of women. This is evident from the observation of the cases of fistulæ especially, which one sees in the ordinary run of cases in a large hospital for women. Formerly fistula between the bladder and vagina was one of the commoner accidents of childbirth, while vesico-uterine fistula was seen occasionally. Now recto-vaginal fistula and lacerated cervix and perineum are the injuries most often met with. The reason for this change is obvious. In the earlier days of Marion Sims and Emmet, patients were being constantly received at the New York Woman's Hospital from the more distant parts of the country, who, having been in labor several days with the child's head wedged in the pelvis, were at last delivered naturally or artificially with the result that the whole anterior vaginal wall and lower wall of the bladder had sloughed away from pressure. In some cases even the anterior lip of the uterus was also destroyed, so that the urine poured from the bladder into the uterus and thence into the vagina. But in those cases of long delayed-labor the perineum was rarely torn, and recto-vaginal fistula, which is generally a result of rupture of the perineum high up, was comparatively rare. The obstetrical fault of those times was leaving the patient undelivered too long. The obstetrical fault of the present day is the termination of the confinement altogether too soon. In a natural labor the child's head comes down and goes back, comes down a little further and goes back again, and so on many times before the perineum is stretched enough to allow the head to pass without rupturing the perineum; but in these days the forceps are used, it is to be feared, too often to save the doctor's time, but at the expense of great inconvenience and suffering to the woman afterward. In some of the worst cases of rupture of the perineum that have come under the writer's notice the accoucheur has been a young man who has been goaded to desperation by the taunts and threats of the old women who are so often to be found present on these occasions, and who draw invidious comparisons between him and other doctors who confined Mrs. Somebody-else in one hour, with her first child. In other cases the patient and all those around her keep asking the doctor if he can do nothing for her, when the head is not yet passed though the *os uteri*. In one case, however, which came under the writer's notice, there was no excuse for the doctor's excess of zeal. The lady, who was pregnant with her first child, was at a little family gathering until nearly eleven p. m., when on reaching home she was taken with the first pain. Not having much experience, she sent for the doctor, so that he might not be out of the way when wanted. The latter arrived in haste with his bag, and at once commenced rapid dilatation of the uterus, or what might be called accouchement force. By two o'clock in the morning he had dilated it enough to put on the forceps, and from this time until six a. m., the husband stated that he applied them thirty-six times, by which time the patient was unconscious, although no anæsthetic had been employed, and the doctor fell back into a chair, saying he was exhausted and could do no more. Another doctor was then called, who found the cervix and perineum frightfully lacerated. The patient was delivered and a month or two later the writer repaired her tear, which extended into the rectum.

Most of the cases of recto-vaginal fistulæ which have come under the writer's notice were the result of such an injury, which had been repaired at the time or soon after, but in which union had not been obtained at the upper end of the tear. The worst case of this kind that the writer has ever seen was a French-Canadian woman, who had been delivered of her first baby at Worcester, Mass. Her accoucheur had sewed up a tear which extended more than half way up to the cervix in the recto-vaginal septum. The front part of the perineum healed very nicely, but at the top of the tear where the septum was very thin, the stitches cut out, leaving an opening the size of a quarter dollar through which her motions all passed. The woman became an object of disgust to her husband and friends, and, on the advice of a sister residing in Montreal, she came here. She was taken in hand by a good surgeon, who freshened the borders of the fistula which were very fibrous, and sewed them together with silk sutures. On the second or third day wind and fæces came through the vagina as before. This surgeon showed great determination of character by repeating the operation four times, always with the same result, the fistula growing bigger each time. The poor woman was now discouraged, but soon after came under the writer's care. After thoroughly preparing her by cleaning out the bowels with castor oil, and after thorough asepsis of the parts, the perineum was cut through as nearly as possible, so as to reproduce the original injury, thus causing a wound of the septum extending from the skin up to the top of the fistula, which had been made larger and larger by each process of freshening of the edges which it had undergone so many times. The vagina was then carefully separated from the rectum until a point rather more than half an inch above the utmost limit of the fistula was reached. The tear in the rectum was now repaired by about fifteen interrupted catgut sutures, which were tied as soon as they were introduced, the knots all being turned into the rectum. When nearing the lower end of the gut, care was taken to get a good hold of the ends of the sphincter, the ends of which were carefully freshened.

The vagina was then treated in the same way, so that two perfect tubes were obtained, and the condition of the perineum appeared the same as if a flap-splitting operation had been performed. Silk-worm gut sutures were now passed with a very long curved Peaslee needle from one side of the perineum to the other, taking care to go above the highest part of the rectal and vaginal stitches, but always keeping between the two canals. When these silk-worm gut sutures were tied there was a very strong perineal body, and the fistula was thoroughly closed by three different layers of sutures, so that sterilized milk injected into the rectum did not come through. The patient was ordered to subsist on unlimited quantities of beef tea, chicken broth, and other fictitious substitutes for food, for three days, and after that she was allowed water gruel, well-boiled and strained. She was strictly forbidden milk in any shape or form on account of the large, hard motions it almost always produces. All went well for three days, when the patient suddenly felt a gust of wind from the vagina. The patient knew by experience that the repair had given away again. She said that she felt sure that if that wind could be prevented from accumulating in the rectum, the operation would be successful. This the writer determined to do the next time by placing a tube in the rectum and leaving it there until the union had time to become firm. The stitches were all removed and the surfaces freshened up and washed with bichloride solution, and the same operation was repeated as before. A piece of soft rubber tubing with a cross-piece passed through the end of it was introduced and held there by the cross-piece. This time the operation was a complete success, great quantities of wind passing out of the tube and good union being obtained.

These two points are of great importance, namely, always to insert a tube in the rectum to prevent the latter being distended with wind, and to forbid the use of milk in any shape or form for ten days. When the bowels are first moved it should always be by enema and never by cathartics, which might drive a large hard mass of fæces against the still delicate line of union. The catgut drops into the rectum in about three weeks, while the silk-worm gut is removed in from ten to twenty days.

Another case to which the writer was called in consultation with Dr. Haldimand, of this city, is of interest from the fact that it was what might be called the primary closing of a fistula. The patient had been delivered three or four days before of a very large child, and having a very firm and small perineum, the latter was ruptured more than half way up to the cervix. The accoucheur at once repaired the perineum by sewing from the top of the tear down to the perineum. Much to his disappointment, on the third or fourth night, he was summoned by the nurse, who informed him that the patient had had a large motion through the vagina. He called upon the writer, with whose assistance the tear was repaired in the same manner as above described, a tube being inserted in the cross-piece to hold it in. Great care was exercised in disinfecting the raw surfaces before sewing them up. The night on which this was done the temperature was 103 degrees, and the pulse was fast; but much to the physician's delight, the temperature fell the next day, wind passed freely by the tube, and good union was obtained, the patient being up in as short a time and as well as if nothing unusual had happened. In this case the physician deserved great praise for repairing the injury without delay, as many cases of puerperal fever and death are due to infection of the open lymphatics of the perineum, deaths which would be prevented if all injury in this locality were promptly repaired.

Small fistula tracts, especially when situated in the perineum near the fouchette, can be closed by setting up inflammatory action and subsequent cicatricial contraction by passing through the fistula a probe covered with a thin layer of absorbent cotton, first wet and then rubbed over a stick of nitrate of silver. One such case came under the writer's care and was completely cured by two or three such applications.

Cases of fistula in ano are tolerably common. One of the longest and most tortuous came under the writer's care in a lady who was incapacitated for work thereby, owing to the discharge and pain. On examining the uterus, it was found to be retroverted and fixed and the ovaries and tubes were lying under it, so that it was decided to repair the fistula first and then to remove the appendages and fix the uterus to the anterior-abdominal wall. The external genitals were first carefully disinfected, as was also the fistula, injecting into it some bichloride solution. A fine gum bougie could be passed into the fistula a distance of nearly three inches, owing to the tortuous nature of the channel, and its location thus being marked out, the perineum was split as in performing Tait's operation. The fistula tract could now be seen somewhat in the form of a corkscrew, and it was quite easy to dissect it out with scissors, just as if it had been a piece of rubber tubing covering the bougie. The sides of the perineum were then brought together with silk-worm gut, making an excellent perineum, and union was obtained by first intention, the stitches being removed in ten days. This lady made an excellent recovery and has never had any trouble since.

Another case of fistula in ano came under the writer's care a year ago at the Montreal Woman's Hospital. It was situated on the right side of the vulva, and was about an inch and a half in depth. The probe could be felt by a finger in the rectum or in the vagina, but there was no communication with either of these passages. On laying it open with the bistoury it was found to be about two inches in length. The writer had had good success in curing fistula in ano in men by pushing a director through the fistula into the rectum, making it a complete fistula, when before it was a blind external one, and then cutting through the sphincter and transversely to its fibres, the wound being then healed from the bottom by keeping it filled with gauze. But this operation is tedious as regards healing, so it was decided to try thorough curetting, disinfection of the fistulous cavity, and allowing it to heal from the bottom. This was done a year ago at the hospital, but was not successful, for a year later, it was in the same condition. A second operation was then performed as follows: The fistula was thoroughly opened up, and the fistulous tract being clearly seen, the latter was picked up with a hook and dissected out with scissors. All sinuses were thoroughly curetted and washed out with bichloride. Deep silk-worm gut sutures were passed from one side to the other, which, being tightened, the whole raw surface of the fistula was brought into accurate apposition. The result was very satisfactory. The fistula closed by first intention and the woman is now free from pain and moisture about the anus. It seems to be the general opinion that the best way to treat a fistula is to cut the fistulous tract out and sew the surfaces together with either several rows of buried catgut or other animal ligature, or a single row of silk-worm gut. The result in these two cases was much more satisfactory than in any of the cases where the sphincter was cut across and allowed to heal by granulation. It is a wise precaution in all these cases to thoroughly stretch the sphincter ani at the time, so as to diminish lateral traction on the apposed surfaces.

*250 Bishop Street.*

